# Assessing the potential of wearable health monitors for health system strengthening in low- and middle-income countries: a prospective study of technology adoption in Cambodia

**DOI:** 10.1093/heapol/czac019

**Published:** 2022-03-09

**Authors:** Marco Liverani, Por Ir, Pablo Perel, Mishal Khan, Dina Balabanova, Virginia Wiseman

**Affiliations:** Department of Global Health and Development, London School of Hygiene and Tropical Medicine, 15-17 Tavistock Place, London WC1H 9SH, UK; School of Tropical Medicine and Global Health, Nagasaki University, 1-12-4 Sakamoto, Nagasaki 852-8523, Japan; Faculty of Public Health, Mahidol University, 420/1 Rajvithi Road, Bangkok 10400, Thailand; National Institute of Public Health, Street 289, Phnom Penh, Cambodia; Department of Global Health and Development, London School of Hygiene and Tropical Medicine, 15-17 Tavistock Place, London WC1H 9SH, UK; Centre for Global Chronic Conditions, London School of Hygiene & Tropical Medicine, London, UK; Department of Global Health and Development, London School of Hygiene and Tropical Medicine, 15-17 Tavistock Place, London WC1H 9SH, UK; Department of Global Health and Development, London School of Hygiene and Tropical Medicine, 15-17 Tavistock Place, London WC1H 9SH, UK; Department of Global Health and Development, London School of Hygiene and Tropical Medicine, 15-17 Tavistock Place, London WC1H 9SH, UK; The Kirby Institute, University of New South Wales, Sydney NSW 2052, Australia

**Keywords:** Cambodia, digital health, health wearables, mHealth, non-communicable disease, cardiovascular disease

## Abstract

Wearable health monitors are a rapidly evolving technology that may offer new opportunities for strengthening health system responses to cardiovascular and other non-communicable diseases (NCDs) in low- and middle-income countries (LMICs). In light of this, we explored opportunities for, and potential challenges to, technology adoption in Cambodia, considering the complexity of contextual factors that may influence product uptake and sustainable health system integration. Data collection for this study involved in-depth interviews with national and international stakeholders and a literature review. The analytical approach was guided by concepts and categories derived from the non-adoption, abandonment, scale-up, spread, and sustainability (NASSS) framework—an evidence-based framework that was developed for studying health technology adoption and the challenges to scale-up, spread and sustainability of such technologies in health service organizations. Three potential applications of health wearables for the prevention and control of NCDs in Cambodia were identified: health promotion, follow-up and monitoring of patients and surveys of NCD risk factors. However, several challenges to technology adoption emerged across the research domains, associated with the intended adopters, the organization of the national health system, the wider infrastructure, the regulatory environment and the technology itself. Our findings indicate that, currently, wearables could be best used to conduct surveys of NCD risk factors in Cambodia and in other LMICs with similar health system profiles. In the future, a more integrated use of wearables to strengthen monitoring and management of patients could be envisaged, although this would require careful consideration of feasibility and organizational issues.

**Key messages**

- This study explored opportunities for, and challenges to, the adoption of health wearables for health system strengthening in a low- and middle-income country.
- The study was conducted in Cambodia, considering stakeholders’ views about the complexity of contextual variables that may influence technology uptake and sustainable health system integration.
- The research findings indicate that health wearables could be used in the short term to conduct surveys of NCD risk factors in LMICs, delivering key information to the health sector that can be used to build richer profiles of population health.
- In the future, wearables could also be used to enable the telemonitoring and management of patients with cardiovascular disease or diabetes, although this application would entail significant feasibility issues.

## Introduction

Non-communicable diseases (NCDs) contribute 73.4% of total deaths worldwide, with a disproportionately high burden in low- and middle-income countries (LMICs) (Roth *et al.*, 2018). In recognition of this, global guidelines to improve the prevention and early detection of NCDs have been developed, such as the World Health Organization (WHO) package of essential NCDs (PEN) interventions (WHO, 2020a). In many countries, however, health system response to NCDs remains a significant challenge (WHO, 2020b). For example, even if care is provided free of charge, travelling times and costs may discourage patients to visit health facilities for regular check-ups, especially those living in remote areas (Geldsetzer *et al.*, 2019). In addition, health information systems (HISs) in LMICs are often unable to record and analyse the breadth of data and information that is needed to assess risk factors for NCDs (Echouffo-Tcheugui *et al.*, 2018). Therefore, crucial is the identification of alternative methods and technologies that can be used to improve monitoring and case management (Hearn *et al.*, 2019).

Wearable health monitors such as smartwatches and smartbands are a rapidly evolving technology that may offer new opportunities for strengthening health sector capacities to address NCDs. Boosted by the commercial success of fitness trackers such as Fitbit and Apple Watch, many wearable devices have been released in recent years, equipped with increasingly sophisticated biosensors, although large variations in accuracy and reliability exist between different products (Piwek *et al.*, 2016). Today, consumer health wearables can capture a wide range of biometric data including heart rate, oximetry, sleeping patterns and mobility, while clinical-grade devices have more advanced features such as blood pressure, biomarkers for blood glucose and hydration levels (Kim *et al.*, 2019). Unlike conventional diagnostic tools, these devices are non-intrusive and can be worn as people follow their daily routines at home or work. As such, they enable continuous real-time monitoring of vital body functions and behaviour related to physical and mental health outcomes, providing greater ecological validity compared to a laboratory setting. In addition, wearables can be designed and adapted to suit diverse applications and contexts.

For these features, wearables have gained increasing appeal in health research, from their use in ‘deep data’ clinical studies to large surveys of population health (Izmailova *et al.*, 2018; Hicks *et al.*, 2019; Ong *et al.*, 2019). For example, Project Baseline is an ambitious research programme involving Google, Stanford University, Duke University and the American Heart Association, which aims to track the health status of 10 000 people in the USA (Arges *et al.*, 2020). In addition to health surveys, blood tests and genome analysis, participants are provided with a study watch, which tracks heart rate and electrocardiogram (ECG), electrodermal activity and inertial movements. Similarly, Singapore’s government has partnered with Fitbit to gather information on the health and behaviour of Singaporeans, implementing the first major integration of a wearable device into a national public health programme (HIS, 2021).

Along the same lines, health wearables could be used to collect data on risk factors for major NCDs in LMICs, providing key evidence to inform policy and planning where the evidence gaps are wider. These devices could also be used to monitor chronic patients with hypertension or diabetes living in remote areas, linking them with the health system for improved disease management (Dunn *et al.*, 2018). During the COVID-19 pandemic, as many patients could not access regular care, the value of telemonitoring has been further highlighted (Monaghesh and Hajizadeh, 2020). Despite this potential, however, there are many barriers to the introduction and scale-up of digital health in LMICs (Opoku *et al.*, 2017; Wallis *et al.*, 2017; van Olmen *et al.*, 2020). In addition to the technology itself, successful implementation requires a detailed assessment of health system resources and readiness (Shaw *et al*., 2018).

In the study presented here, we examined the potential of health wearables for health system strengthening and associated challenges within the country context of Cambodia. After decades of conflicts, from the early 1990s health service delivery in the country has gradually improved through reforms and infrastructure development (Liverani *et al.*, 2018). These efforts, combined with the effects of steady economic growth, have contributed to better health outcomes. However, capacities for the prevention and management of NCDs are still quite limited (Jacobs *et al.*, 2017), despite NCDs are estimated to account for 64% of all deaths in Cambodia and cardiovascular diseases are responsible for the largest proportion (24%) of premature deaths (WHO, 2018a). In view of this, we examined opportunities for, and potential challenges to, the introduction of health wearables for the prevention and control of NCDs, considering the complexity of contextual variables that may influence technology uptake and sustainable health system integration.

## Methods

### Research design

We used concepts and categories derived from the non-adoption, abandonment, scale-up, spread, and sustainability (NASSS) framework—an evidence-based framework that was developed for studying health technology adoption and the challenges to scale-up, spread and sustainability of such technologies in health service organizations (Greenhalgh *et al.*, 2017). NASSS provides a comprehensive framework to support systematic assessments of health care innovation, incorporating various perspectives on health and illness, technology adoption, organizational change and health service design. Considering the health system focus of our investigation and the type of technology, we modified some elements in this framework to ensure a better fit with our study and the research setting. We defined ‘adopters’ as encompassing not only staff, patients and/or their caregivers but also citizens at large since health wearables can also be used for the prevention and monitoring of NCDs in healthy or asymptomatic individuals. Furthermore, in the last dimension, we focused on ‘sustainability’ (rather than ‘embedding and adaptation over time’ in the original framework), understood as the extent to which the technology can be maintained in the long term. This change was made to ensure we captured the challenges around financing and local ownership of health technologies in LMICs.

The full description of research domains is presented in Table 1. To explore these domains, we conducted qualitative interviews with national and international stakeholders, selected among those who could bring expertise and insights on NCDs in Cambodia and/or had been involved in mHealth programmes in the country. For the purpose of demonstration with stakeholders, we used a watch-type consumer health wearable produced by a company in Shenzhen, China, which could measure blood pressure, heart rate, sleeping time, steps taken and calories. In addition to the interviews, various documents were identified and reviewed at different stages in the research process to provide contextual information and further insights (particularly for domains 1, 6 and 7).

**Table 1.** Research domains adapted from the NASSS framework

1. The health issue	The nature of the health problem and the health system response
2. The technology	The type of technology, including its material properties, accuracy and availability
3. The value proposition	Whether a new technology is worth in the first place and for whom it generates value
4. The adopters	Staff and end users who may accept or refuse the technology or find they are unable to use it
5. The organizations involved	The capacity of the organizations involved to integrate and support the technology
6. The wider context	(1) The infrastructure, (2) the policy and regulatory framework and (3) sociocultural factors that may influence technology adoption
7. Sustainability	The extent to which the technology can be maintained in the long term

### Interviews

The semi-structured interviews were conducted between March and October 2019 with managers in the Ministry of Health in Cambodia, local and international non-governmental organizations (NGOs) working closely with hypertensive and diabetic patients in the communities, and representatives of multilateral organizations based in Cambodia such as the WHO. An initial set of stakeholders was identified using the professional network of the researchers involved in this study. Others were selected by snowball and purposive sampling. The interviews with stakeholders lasted about 1 h and were conducted face-to-face at their workplaces by the first and/or the second author in the capital Phnom Penh. The interviews revolved around the following topic areas: (1) challenges to NCD prevention and control in Cambodia, particularly in relation to the surveillance and monitoring of risk factors; (2) previous experiences with mHealth technologies; and (3) views and suggestions about the potential use of health wearables for health system strengthening in Cambodia. Depending on the expertise and experience of each informant, the interview schedule was modified to capture different elements in the technology adoption framework, including organizational issues, technical challenges and opportunities, acceptability, sustainability, governance and policy aspects. After each interview, the topic guide was refined in light of emerging findings and participants’ feedback. All interviews were conducted in English. Where consent was given, interviews were audio recorded and then transcribed by the first author; otherwise, extensive notes were taken during and after the meeting. The study was approved by the Ethics Committees of the authors’ institutes. Information on the aims and objectives of the research project was provided to all participants and informed consent was obtained from all of them prior to being interviewed. All data were anonymized and stored in a secure offline archive.

### Literature review

We searched the databases Google Scholar, PubMed and Web of Science using the terms ‘non-communicable disease*’, ‘NCD*’, ‘cardiovascular disease*’, ‘hypertension’, ‘diabetes’, ‘digital health’ or ‘mHealth’ in combination with ‘Cambodia’. We restricted our search to articles in English that were broadly associated with NCDs (including disease burden, national policy and health system response) and mHealth programmes in Cambodia. Additional documents were sourced during the interviews or from manual searches of websites belonging to relevant organizations such as the Ministry of Health (MOH) in Cambodia.

### Qualitative data analysis

Thematic content analysis of the information gathered from the interviews and the literature review was guided by the NASSS conceptual framework described above. Within each research domain, open coding was used to enable a broader reading of data and the identification of sub-themes through an inductive and iterative approach. The first author coded the transcripts using the qualitative data analysis software NVivo 12, while regular discussions were held with the team members to verify the interpretation of findings. The Consolidated Criteria for Reporting Qualitative Research guidelines (COREQ) were used to ensure comprehensive reporting of the data collection and analysis procedure (Tong *et al*., 2007).

## Results

In total, we interviewed 18 informants, including 7 managers within the MOH and related bodies, 3 directors of local non-governmental organizations (NGOs), 4 managers of international NGOs based in Cambodia and 4 officers from multilateral organizations in Cambodia including the WHO and the World Bank (Table 2). In the sections below, we present the main study findings, organized according to the seven dimensions adapted from the NASSS framework (Table 1). Anonymized citations are included to illustrate key points and referenced by the unique identifier BIOS-*n*. Findings from the literature review are integrated into the main text to explore specific points or provide contextual information.

**Table 2.** Participants by type of organization and identifier

Organization	Unique identifier
Ministry of Health	BIOS 02,04, 08, 09, 13, 15, 18
Local NGOs	BIOS 01, 14, 17
International NGOs	BIOS 03, 05, 05, 07
International organizations	BIOS 10, 11, 12, 16

### The health issue

In the past decade, the government of Cambodia has fully recognized the growing burden of NCDs. In 2013, the MOH approved the first National Plan for the Prevention and Control of NCDs amidst concerns that Cambodia would face a ‘tsunami of additional NCD patients in the coming years’, threatening to overwhelm the health system and to exacerbate health inequities (MOH, 2013). This commitment was renewed in the 2018–2027 plan, which focuses on the prevention and early detection of cardiovascular disease, cancer, chronic respiratory diseases and diabetes (MOH, 2018). Within this policy framework, several NCD interventions have been implemented, including chronic disease outpatient clinics at provincial hospitals, peer educator networks for diabetes and hypertension and essential NCD services at health centres, based on the WHO standard PEN.

Despite some progress, a consensus emerged during the interviews that capacities for cardiovascular risk assessment and diabetes management are still inadequate throughout the country, particularly at the primary health care level where routine monitoring of risk factors is unusual. Similarly, a recent study found ‘a considerable time lag before people are diagnosed with their conditions, frequently a result of failure of health providers to measure blood pressure or screen for blood sugar levels’ (Jacobs *et al.*, 2017).

Lack of reliable data on NCDs was identified as another important health system gap. As several informants pointed out, the health information system (HIS) in Cambodia was developed during the 1990s, when infectious diseases were the main public health concern in the country (BIOS01, BIOS09, BIOS11):

*One problem is that the health information system in Cambodia – and the health system in general - was designed to address acute care needs when the patient turns out sick (…) but little investment has been made so far to strengthen the case management system for chronic care, NCD prevention, and risk factors. The information system was not designed for that (…) it should be completely redesigned to account for the epidemiological transition* (BIOS01).

Recognizing this gap, a new Patient Management and Registration System (PMRS) is being developed to record electronic data on NCD indicators (e.g. blood pressure, heart rate and body mass index) during routine visits at public health facilities (BIOS15). According to one informant, however, implementation has been slow and incomplete:

*The PMRS doesn’t work so well and doesn’t really capture NCD data (…) in theory we need 30 NCD indicators, but there are only a few in the PMRS; in addition, the national health information system and the PMRS still run in parallel, they are not integrated* (BIOS09).

The availability of other forms of evidence on NCDs is also scarce, partly due to a lack of interest among donors and funding agencies (Goyet *et al.*, 2015; Liverani *et al.*, 2018). In 2010 and 2016, two surveys of NCD risk factors were conducted, based on the WHO STEPwise approach to Surveillance (STEPS). In addition, a handful of studies of NCD prevalence and services have been published in recent years (Gupta *et al.*, 2013; Bigdeli *et al.*, 2016; Jacobs *et al.*, 2016; Chhoun *et al.*, 2017; Jacobs *et al.*, 2017; Kobashi *et al.*, 2020). However, the evidence base remains thin, particularly on risk factors. As one Cambodian doctor pointed out: ‘We really don’t know much about NCDs and health behaviours… Do Cambodians work out? Do they sleep well? How is hypertension? We lack reliable data’ (BIOS18).

### The technology

Considering these health system constraints, we discussed with stakeholders the potential of wearables to improve the monitoring of NCD risk factors and/or case management. As mentioned in the methods section, a watch-type consumer health wearable was used for the purpose of demonstration with stakeholders. During the interviews, many participants found this model attractive due to its multiple functions, smart design and user-friendly graphical interface. However, the director of a local NGO with several years of experience in the implementation of mHealth projects in Cambodia stressed:

*It is difficult to make public health decisions involving these technologies because the [technology] landscape changes constantly* (BIOS01).

Other participants had concerns about the accuracy of measurement—particularly the blood pressure monitor—resilience and battery life (BIOS14, BIOS17).

In addition, an expert in digital health solutions highlighted the need for an open application programming interface (API) that would enable adaptation of the device to the Cambodian context, including language:

*If the watch has an API, it is open to development of third-party software. So, the device can be customised. However, these devices are manufactured by private companies… We cannot really modify them* (BIOS05).

### The value proposition

Participants in our study were interested in health wearables and highlighted their potential value to address different health system challenges. As described, cardiovascular risk assessment and management to prevent heart attacks and strokes are still inadequate in Cambodia, especially in rural areas (Jacobs *et al.*, 2017). In these settings, as two participants suggested (BIOS08, BIOS13), health wearables could be given to patients with chronic heart disease—such as heart failure—to provide continuous monitoring of blood pressure, heart rate and ECG. In turn, user data could be shared with the local health centre or the district hospital who could send the patient feedback on disease self-management or reminders to visit the health facilities. One officer within the MOH pointed out that ‘this would bring the health system to the patient’ and therefore would be useful to people living in remote areas (BIOS15). Other participants suggested the device could be designed to send an automatic emergency alert to the district health office in the event of anomaly (such as detection of atrial fibrillation), as seen in consumer wearables such as the Apple iWatch. Reflecting on these potential applications, an international consultant envisaged a ‘new model of care’ ushered in by these new technologies:

*Well, you know… you could think of a new model of care in which nurses sit in offices and monitor these semi-continuous flow of data… and for emergency cases you could send a nurse with first aid to see the patient* (BIOS16).

During the interviews, participants also mentioned that wearables could be used to support and improve data collection in surveys of risk factors for NCDs:

*These watches could be used to monitor whether the level of physical activity is increasing or decreasing in Cambodia (…) We don’t know much about this. There is little evidence available, so we cannot make decisions* (BIOS09).

Another participant stressed that the watch would offer advantages over standard data collection methods such as the questionnaires in the WHO STEPS (BIOS04). As he explained, STEPS is based on self-reported measures of physical (in)activity and other behaviours, which are prone to biases. In contrast, the watch would enable the collection of reliable data in a natural setting over an extended period of time.

The potential value of health wearables for health promotion and behavioural change was also highlighted, although participants were more sceptical about this prospect. For instance, a technical officer of an international organization based in Cambodia said:

*The watch alone is not enough… you need other things. Fitbit, for example, sends users a weekly report by email; this is a good system, but you need either a phone app or an email account… this might be difficult to achieve in Cambodia* (BIOS11).

### The adopters

As noted above, most stakeholders recognized the potential value of health wearables for health system strengthening. However, our analysis identified various obstacles to technology uptake, depending on the type of adopter (health workers, patients or caregivers) and the intended use of the health wearables. From a health sector perspective, participants mentioned that the introduction of wearables for case monitoring and case management would require extensive training of health workers and a rethinking of established roles and practices, posing additional health system challenges (BIOS17, BIOS18). Another participant highlighted the importance of ‘human connection’ between health providers and patients in the communities, arguing that peer educators had a greater impact on self-management of hypertension and diabetes in comparison with eHealth solutions (BIOS01). In addition, several participants pointed out that health wearables would be suitable to young urban residents but uptake in other population groups such as farmers and the elderly could be challenging for practical and cultural reasons:

*Middle-aged people would need something really low-tech…people who were born before the Khmer Rouge period don’t really use technology in Cambodia, but the young people pick it up fast* (BIOS06).

*Many farmers who work in the field don’t like wearing a watch because it could be inconvenient and there is a high risk of damaging it* (BIOS08).

Lastly, some participants expressed concerns about the ability of patients to interpret data and trends. Notably, the director of a local NGO stressed that ‘maybe there is a patient with symptoms of stroke, but he will not go to the doctor because the health watch does not detect any problems, or the user cannot understand… this is dangerous’ (BIOS14).

### The organizations involved

Over the past decade, the MOH in Cambodia has been open to innovation and the piloting of mHealth solutions for different health issues, including malaria control (Ngor *et al.*, 2018), maternal and newborn health (Huang and Li, 2017) and family planning (Smith *et al.*, 2013). Furthermore, when we conducted this project, a digital health working group with advisory functions and WHO support had just been formed in the MOH. As one stakeholder pointed out, this provides a favourable ground for the introduction of digital health solutions in Cambodia (BIOS16). Within this framework, for example, the MOH and international partners were piloting the HeartCare package, a user-friendly software developed by WHO to simplify and standardize prediction and management of cardiovascular risk at primary care facilities (WHO, 2018b).

Other participants were more cautious about the readiness of the national health system for digital health innovation. First, as mentioned earlier, participants stressed that the HIS is not able to fully integrate large volumes of NCD data yet, including those collected using the HeartCare package (BIOS16, BIOS18). Second, there was agreement that human resource capacities within the MOH are still too limited to manage complex digital projects, including data processing and analysis (BIOS03). Furthermore, it was noted that record keeping is often neglected at community health centres due to a shortage of health workers and competing tasks. Thus, any additional burden in terms of data collection and monitoring would be difficult to take on (BIOS19).

### The wider context

Meaningful and effective implementation of digital health interventions is a complex task which requires resources and planning beyond the health sector, including nationwide internet access, an enabling policy environment and regulatory framework and conducive sociocultural factors, considered in turn below.

#### Infrastructure

Reflecting on the resources needed for the uptake of health wearables, one local stakeholder noted:

*Coverage of internet and electricity are improving… phones are also becoming cheaper… We are more dependent on technology now… so, this is a good time for developing this kind of eHealth applications…* (BIOS09).

Nonetheless, recent surveys have found wide gaps in access to these resources, particularly in remote rural areas. In 2018, only 40% of the national population in Cambodia had individual internet access, with wide gaps between urban and rural areas (UN, 2020). Similarly, in 2017 less than a third of health centres had a computer (MOH, 2017).

#### Policy and regulatory framework

The introduction of digital health solutions would also require appropriate regulations to ensure the protection of user data. In 2019, the government of Cambodia enacted the Law on Electronic Commerce (‘e-commerce law’), which mandates any business that stores personal information in electronic format to ensure that the data is reasonably protected from loss or unauthorized access, use, alteration, leaks or disclosures (GOC, 2019). While this law is an important step to increase data protection in Cambodia, it focuses on commercial use of data, and it is unclear whether it can be applied to the public sector. Moreover, it was noted that no legal provisions address potential data breaches or data breach notifications in the health sector. As one informant pointed out, it is not clear how this information can be stored securely and who can access it (BIOS18). Another issue is the regulatory approval of health wearables for public health use. At the time of writing, some devices had received approval by food and drug administrations in other countries, but not in Cambodia.

#### Social/cultural factors

Local stakeholders believed the watch could be attractive to the tech-savvy young generations, but less appealing to senior citizens who tend to see the classic wristwatch as a status symbol. Three participants also noted that the concept of health self-monitoring is not well established in Cambodia since the elderly are often taken care of by their children (BIOS04, 05):

*The trend in Cambodia is that the elderly are taken care by their children, who also give them medicines. The elderly often don’t even know what medicines they must take and their hospital appointments. So, caregivers should also be able to access feedback and messages from the watch* (BIOS04).

One participant, however, recognized that a process of generational change is under way in the country whereby more people than before live alone:

*In five or ten years we might have more people who are in a self-care situation and the young generation now will be the next generation with chronic diseases. The health watch will be useful to them* (BIOS04).

### Sustainability

Health system integration and sustainability are key issues in technology adoption, particularly in LMICs where human resources and budget are more limited. In Cambodia, sustainability and national ownership of donor-driven health technologies have been recurrent challenges:

*Different donors support different health technologies. But the donors also have their own agendas and protect what they have developed… they often do not allow the integration of these technologies into the existing system* (BIOS18).

In addition, a health manager within the MOH was frustrated there was a lack of coordination between different mHealth initiatives funded by different donors and no international or domestic budget to support scale-up beyond pilot projects:

*Today, there are many new mHealth projects on NCDs including two projects financed by China. There are so many funders, so many projects! But there is little coordination…This was an issue with infectious diseases and now we have the same problem with NCDs* (BIOS15).

## Discussion

In our study there was a consensus that substantial gaps remain in the ability of the health system in Cambodia to address NCD challenges, from prevention and monitoring to case management and follow-up. Considering these gaps, stakeholders discussed three potential public health applications of wearables for the prevention and control of NCDs: to support health promotion; to monitor patients with cardiovascular disease and link them with the health sector and to conduct population surveys of NCD risk factors. Most participants were positive about these prospects and willing to support pilot projects to further assess their feasibility and impact. Furthermore, our investigation found that recent developments in Cambodia could facilitate technology adoption, including increasing internet access, the diffusion of mobile technologies and a favourable health policy framework.

Despite these opportunities, key challenges to sustainable technology uptake were identified across different domains, depending on the intended use (Table 3). First, the use of wearables in public health programmes (particularly for case management and follow-up) would require substantial investment in human resources. Indeed, stakeholders emphasized that successful programme implementation can only be achieved with a motivated health workforce, trained and prepared to embrace the innovation. However, as found in other studies of mHealth in LMICs (Feroz *et al.*, 2018; Osei and Mashamba-Thompson, 2021), work overload, competing tasks and lack of familiarity with digital health technologies may affect technology adoption and appropriation in the long term.

**Table 3.** Potential uses of wearables and main implementation issues

Potential use	Main implementation issues
Health promotion—Deliver motivational messages to encourage a healthier lifestyle	- Technology usability and durability - Lack of internet access for some groups - Cultural acceptability, particularly among the elderly - Financing
Case management—Follow-up and telemonitor the health status of patients with chronic conditions and send medication and check-up reminders	- Technology usability and durability - Technology reliability - Lack of internet access for some groups - Health system integration - Limited health workforce capacity - Cultural acceptability, particularly among the elderly - Financing - Privacy protection
Health surveys—Collect data on health status and behaviour as part of surveys of risk factors for NCDs	- Technology reliability - Lack of internet access for some groups - Privacy protection

Second, the research findings indicate that the benefits from technology uptake would be unevenly distributed among the population. A watch-type health wearable may be suitable to young urban residents, but uptake in other population groups such as farmers and the elderly could be difficult for both practical and cultural reasons. Thus, health wearables may reproduce and even reinforce existing inequities in access to care (Liverani *et al.*, 2020), if the needs of different population groups are not considered in technology design and programme implementation (Smith *et al.*, 2015; Babatunde *et al.*, 2021). In this respect, we should also note that a survey of technology acceptance published elsewhere found that willingness to pay (WTP) for the same technology in Cambodia was an important barrier to product uptake, especially amongst poorer populations (Liverani *et al.*, 2021). While the average WTP was high (US$11.4) relative to a gross national income per capita of US$1530 (World Bank, 2019), many participants in the lowest socio-economic group were willing to pay only a fraction of the estimated market value of the sample watch used in the survey (about US$30) and less than two-thirds were willing to buy the watch.

Third, health system integration of user data would pose substantial technical challenges. The HIS in Cambodia is not yet able to incorporate and process routine electronic data on NCDs and associated risk factors. As in many other LMICs, investments in the public health sector have prioritized other health system functions ahead of information technologies, resulting in a lack of infrastructure to support mHealth services (Feroz *et al.*, 2018). Furthermore, current initiatives to evaluate digital health solutions (such as the working group on digital health) are not yet institutionalized and enshrined in national policy, providing little guidance to inform system integration of pilot projects (Wilson *et al.*, 2014).

Lastly, the case of Cambodia highlights the importance of sociocultural factors in technology adoption. As recent sociological studies pointed out, the so-called ‘wearable revolution’ is characterized by ‘a shift of responsibility from the medical expert to the tracking technology and to the individual’ (Fotopoulou and O’Riordan, 2016). This new model of care is premised on an individualistic approach in which ‘digitally engaged patients’ are expected to manage their own preventive health efforts (Lupton, 2013). Even if wearables can be part of a wider health care system that also involves human interactions, the concept of self-care remains central to the uptake of this technology. In Cambodia, this may be at odds with traditional culture and norms which emphasize the collective, social dimension of caring and disease management, particularly for the elderly. However, these considerations may lose relevance in the future, as the next generation of Cambodians is shifting to a more individualistic lifestyle.

### Recommendations for policy and practice

Based on our findings, wearables could be used in the short term to support and complement health surveys of risk factors for NCDs in Cambodia. In this application, wearables would provide continuous measurement of behaviour and biomarkers as subjects follow their daily routines, delivering key data to build richer health and epidemiological profiles for the prevention of NCDs, which can, in turn, be used to inform the national strategy on NCDs. In comparison with other applications, the use of wearables in health surveys would be less demanding in terms of human and material resources, requiring a small research team and a limited number of health wearables.

In the long term, an innovative model of care could be envisaged where wearables are used for telemonitoring and management of patients with chronic diseases. In addition to cardiovascular risk assessment, new biosensors are being developed which can provide painless, needle-free and continuous monitoring of blood glucose (Hanna *et al.*, 2020). These sensors have great potential to enhance the quality of life for diabetic patients and their retention in care and would be particularly useful for patients with limited access to health facilities such as those living in remote rural areas or with disabilities. However, this use would be more complex as well as resource and labour intensive, involving substantial organizational challenges for health providers and a rethinking of their established roles and practices.

Regardless of the intended use, accuracy and costs are an important concern across the board. At the time of writing, the Omron HeartGuide, a reliable watch-type blood pressure monitor approved for medical use by the Food and Drug Administration in the USA, costs about US$500. Thus, wide technology uptake would likely require some form of subsidization, unless the cost of medical-grade wearables will fall in the near future or a limited number of devices are purchased by hospitals and provided on rotation to patients for surveillance and monitoring purposes in relatively short periods of time. Another important consideration is that any products for public health use should have an open programming interface to enable adaptation to the local needs and health system. In this respect, initiatives to promote the open sharing of algorithms for sensor-generated measures of health and user interfaces have been launched in recent years (OWI, 2021). This could facilitate the design of technologies for distribution outside mainstream markets in high-income countries and their not-for-profit use in health and development programmes. Finally, the recent adoption of the e-commerce law is an important step towards the protection of electronic data in Cambodia. Yet a more sophisticated regulatory system should be developed to address the complexity of ethical issues involved in the collection and storage of individual health data.

### Study strengths and limitations

To the best of our knowledge, this is the first systematic assessment of the potential of health wearables for health system strengthening in an LMIC. The research findings can be used to inform policy development and further research in Cambodia as in other settings with limited health sector capacities to address the challenges of NCDs. In addition, our study further highlights the value of NASSS as a flexible research framework, which can be adapted and used to conduct not only implementation studies (Abimbola *et al.*, 2019; Kadesjö Banck and Bernhardsson, 2020) but also prospective health system analyses of technology adoption. However, more research work is needed to evaluate the impact of wearables on access to care and health outcomes with formative research on different types of wearables as the user experience may differ. In addition, cost-effectiveness analyses would provide systematic evidence on whether these technologies provide the best value for both patients and the health sector relative to other interventions. Lastly, given the prospective nature of our study, we can only speculate on sustainability issues and technology adoption in the long term.

## Conclusion

This study provides new insights into the public health value of wearables and their potential contribution to the prevention and control of NCDs in LMICs. The research findings indicate that health wearables could be used in the short term to conduct surveys of NCD risk factors, delivering key information to the health sector that can be used to build richer profiles of population health. In a more distant future, wearables could also be used to enable the telemonitoring and management of patients with chronic cardiovascular diseases and diabetes, although this would require careful consideration of significant feasibility issues, including costs, human resources and technical infrastructure. It is hoped our study will stimulate further interest and research in the potential applications of this technology, in Cambodia and other LMICs.

## Data Availability

The anonymised dataset is available upon reasonable request.
